# On the Stability of c-BN-Reinforcing Particles in Ceramic Matrix Materials

**DOI:** 10.3390/ma11020255

**Published:** 2018-02-07

**Authors:** Anne-Kathrin Wolfrum, Björn Matthey, Alexander Michaelis, Mathias Herrmann

**Affiliations:** 1 Fraunhofer IKTS, Fraunhofer Institute for Ceramic Technologies and Systems, 01277 Dresden, Germany; anne-kathrin.wolfrum@ikts.fraunhofer.de (A.-K.W.); bjoern.matthey@ikts.fraunhofer.de (B.M.); Alexander.Michaelis@ikts.fraunhofer.de (A.M.); 2 Institute for Materials Science, Dresden University of Technology, 01062 Dresden, Germany

**Keywords:** cubic boron nitride, c-BN, composites, phase transformation, hexagonal boron nitride, microstructure

## Abstract

Cubic boron nitride (c-BN) composites produced at high pressures and temperatures are widely used as cutting tool materials. The advent of new, effective pressure-assisted densification methods, such as spark plasma sintering (SPS), has stimulated attempts to produce these composites at low pressures. Under low-pressure conditions, however, transformation of c-BN to the soft hexagonal BN (h-BN) phase can occur, with a strong deterioration in hardness and wear. In the present work, the influence of secondary phases (B_2_O_3_, Si_3_N_4_, and oxide glasses) on the transformation of c-BN was studied in the temperature range between 1100 °C and 1575 °C. The different heat treated c-BN particles and c-BN composites were analyzed by SEM, X-ray diffraction, and Raman spectroscopy. The transformation mechanism was found to be kinetically controlled solution–diffusion–precipitation. Given a sufficiently low liquid phase viscosity, the transformation could be observed at temperatures as low as 1200 °C for the c-BN–glass composites. In contrast, no transformation was found at temperatures up to 1575 °C when no liquid oxide phase is present in the composite. The results were compared with previous studies concerning the c-BN stability and the c-BN phase diagram.

## 1. Introduction

There is an ongoing need for materials with high wear resistance for bearings, seals, nozzles, and other wear parts in machines as well as for cutting tools and drawing dies in the metalworking industry [1,2,3]. The last few years have seen the development of diamond-based ceramic materials that can be produced without the application of high pressures [3,4]. CVD (Chemical Vapor Deposition) diamond coatings on SiC have been introduced for sealing applications [5,6]. Diamond-based composites are not suitable for use in cutting tools for ferrous metals or for applications at elevated temperatures due to the chemical instability of diamond under these conditions. Particularly for high-speed metal cutting, ceramic inserts based on Si_3_N_4_/sialon and Al_2_O_3_ materials are used, along with polycrystalline cubic boron nitride (PCBN) cutting tools. The PCBN cutting tools are produced at high pressures and temperatures due to the fact that the cubic BN phase (c-BN) converts to the soft hexagonal modification (h-BN) during densification under near-ambient pressure conditions [3,7,8,9,10].

Attempts to produce highly wear-resistant c-BN-reinforced ceramic materials under low pressure based on Si_3_N_4_, sialon, Al_2_O_3_, and SiO_2_ [11,12,13,14,15,16,17,18,19,20,21] have been made. The availability of fast densification methods such as spark plasma sintering (SPS) has piqued interest in manufacturing and investigation of the properties of these materials. However, only a moderate increase in hardness has been achieved thus far with the addition of c-BN [11,12,13,14,15,16,17,18,19,20,21]. For c-BN volume contents higher than 20 vol %, a decrease in hardness [12,15] and a strong increase in fracture toughness [11,12,13,14,16,17,18,19] have been found. This is mainly due to the weak bonding of c-BN to the ceramic matrix resulting from the formation of h-BN layers at the interface [12,13,14,16,18]. Formation of h-BN at the interface has not been reported for sintering temperatures below 1600 °C in other publications dealing with c-BN/Si_3_N_4_ materials, probably due to the difficulties experienced in detecting these layers by X-ray diffraction (XRD) or scanning electron microscopy (SEM).

Zhang et al. [2] published results regarding the stability of c-BN in different matrix materials. WC/Co. Al_2_O_3_, and Al_2_O_3_/Ni were found to accelerate the transformation of c-BN to h-BN, which took place at approximately 1600 K (1323 °C) in these materials; whereas sialon, mullite, and SiO_2_ were found to retard the transformation, which took place in these matrix materials at approximately 1900 K (1623 °C).

Sachdev et al. [22] reported that this phase transformation preferentially started on single c-BN crystal surfaces and depended on the c-BN crystal size and impurities. Sachdev and coworkers [23] investigated the interaction of different components, including a B_2_O_3_ melt, with c-BN. The crystals were completely covered by the melt. Interaction with a B_2_O_3_ melt resulted in roughening of the surface at 850–900 °C. After 20 h, nearly total decomposition of the c-BN took place, but no new compounds were determined by XRD. The authors proposed the formation of B_x_N_y_O_z_ species.

The transformation was studied via DTA by Sachdev et al. [22]. The findings showed an onset temperature of 900 °C for fine-grained crystals (with a size of 1.5 µm) and 1500 °C for coarser-grained crystals (with a size of 600 µm) [22]. The authors observed fast transformation above 900 °C for the c-BN with small grain size. This powder exhibited a high B_2_O_3_/HBO_2_ content, but the authors did not take this in account. The authors suggested two mechanisms: solid phase transformation and gas phase transport. The experiments were carried out using Pt-crucibles, which could react under these conditions and form platinum borides. This interaction could have an influence on the observed effects.

Based on recent TEM investigations of the c-BN–sialon interface [13] and development of special preparation methods for these materials [12,19,24], it has been shown that even following sintering at 1525 °C, by means of SPS, a hexagonal BN layer forms at the c-BN–sialon interface. The interface is composed of nanocrystalline or sub-µm h-BN particles embedded in an yttrium-rich oxynitride glassy phase [12,13,14,19]. A small yttria-rich layer is always found between the c-BN grains and the newly formed h-BN grains. These results reveal the important role played by the existence of the liquid phase in the transformation. The distribution of the h-BN in the matrix suggests that the transformation is controlled by a dissolution–diffusion–precipitation mechanism [12,13]. In the present work, c-BN grains were embedded in a pure Si_3_N_4_ matrix without any sintering aids, so that no liquid phase is present during sintering, in different oxide glasses as well as boron oxide. The microstructure was analyzed after the different heat treatments at temperatures between 1100 °C and 1575 °C for the purposes of verifying this hypothesis and clarifying the role of the liquid phase during phase transformation.

## 2. Materials and Methods

Cubic boron nitride powder with a mean particle size of 20 µm (Vollstädt Diamant GmbH, Seddiner See, Germany) was used as the starting material. The oxygen content of the starting c-BN powder was 0.07 wt %.

For the purposes of studying the influence of the amount of boron oxide at the c-BN particle surface on the c-BN phase transformation, the c-BN powder was oxidized in the STA (Simultaneous Thermal Analysis) in an alumina crucible in synthetic air at 1000 °C for 5 h with a heating and cooling rate of 10 K/min (Netzsch STA 429), resulting in pronounced B_2_O_3_ formation in the powder (2.37 wt % increase in mass, corresponding to approximately 8 wt % B_2_O_3_). The oxidized c-BN powder and the as-received c-BN powder were then heat-treated in an argon atmosphere (oxygen and H_2_O impurities < 10 ppm) with a heating and cooling rate of 10 K/min at 1550 °C for 20 min in an alumina crucibles in the STA (Netzsch STA 449F1). The heat-treated c-BN powders were analyzed by FESEM (Ultra 55, Zeiss Ltd., Oberkochen, Germany) and Raman spectroscopy (LabRAM HR Raman micro imaging system, laser wavelength 473 nm, Horiba Scientific Ltd., Bensheim, Germany).

To investigate the influence of the availability and the composition of the liquid phase during sintering on the c-BN-stability, the c-BN powder was mixed with pure Si_3_N_4_ powder (SN-E10, UBE), and with Si_3_N_4_ powder containing Al_2_O_3_/AlN and Y_2_O_3_ additives forming a liquid phase during transition. The details are given in the literature [12,13,14,19]. In the present study, the sialon Y_m/3_Si_12−m−n_Al_m+n_O_n_N_16−n_ with m = 0.5 and n = 1 was used. The composition had an excess of 4 wt % Y_2_O_3_. Therefore, it forms a transient liquid, which is consumed during sialon formation, and a permanent liquid phase after full conversion of the α-Si_3_N_4_ powder to α-sialon [12,13,14]. Samples of 20 mm in diameter were sintered with the spark plasma sintering equipment (HHP D25, FCT Systeme Ltd., Frankenblick, Germany) in a vacuum at 1575 °C for 5 min with a heating rate of 50 K/min with a pressure of 50 MPa.

To investigate the influence of liquid oxide phases on the transformation composites of c-BN with three different glass matrices were additionally produced. Table 1 lists the compositions used for the three glass matrices G1–G3.

All the composites contained 30 vol % of c-BN and were produced by wet mixing for 1 h in a ball mill containing agate balls and isopropanol and then drying and sieving (400 µm). The preparation of the glasses G1 and G2 is described elsewhere [26,27]. Glass G3 is an Alkaline earth aluminosilicate glass “8252” from Schott. Glass G2 was very similar to the glasses normally found in Si_3_N_4_ or sialon materials. The other two glasses contained different amounts of B_2_O_3_ and, consequently, different viscosities and stabilities. From each of the three different glass–c-BN powders, approximately 2 g of the mixture was placed in a graphite crucible and compacted by hand with a small graphite punch. The samples were heat-treated in a furnace with an argon atmosphere and graphite heating elements at different temperatures ranging from 1100 °C to 1400 °C and different holding times (see Table 2 and Table 3). After heat treatment and sintering the c-BN–glass composites were cut, embedded in resin, and polished. The final polishing step was performed using ion beam polishing [24] to ensure artefact-free microstructures.

The microstructures of the samples were examined in an FESEM (Ultra 55, Zeiss). Raman spectra were recorded using a Horiba Scientific Ltd. LabRAM HR Raman micro imaging system. The laser wavelength was 473 nm and the laser spot size was 0.85 µm.

Crystalline phases were identified qualitatively using XRD analysis (D8 Advance, Bruker AXS, Karlsruhe, Germany) with CuKα radiation over a two-theta range of 5–90° 2Θ with a step size of 0.02° 2Θ. The h-BN content was quantified for the different heat-treated c-BN–glass composites using TOPAS V5 software (Bruker AXS) with the PONKCS method [28] and the structural informations of c-BN (ICSD No. 42002) and h-BN (ICSD No. 24644). With the method of Scarlett and Madsen [28] it is possible to calculate the content of phases without any structural information. The calibration of the matrix was done by adding a c-BN powder as internal standard in the powderized glass samples (G1–G3), which were heat treated parallel to the corresponding glass-c-BN composites, to calculate the ZM constant [28]. For the quantification of the h-BN/(c-BN + h-BN) content the calibrated set of reflections was used together with the crystallographic structures of c-BN and h-BN.

## 3. Results

### 3.1. Heat Treatment of the Pure and Oxidized c-BN Powders

**Figure 1:**
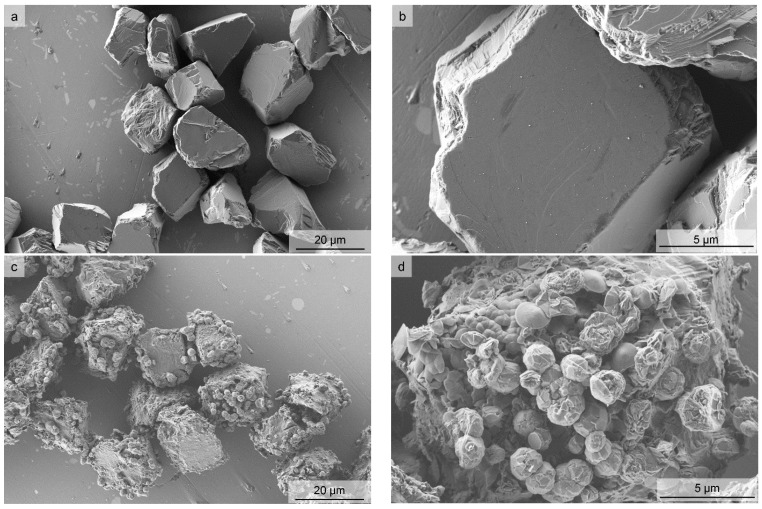
FESEM micrographs of the initial (**a**,**b**) and the prior oxidized (**c**,**d**) cubic boron nitride (c-BN) powders after heat treatment at 1550 °C in Ar.

The as received c-BN powder and the oxidized c-BN powder were heat-treated at 1550 °C for 20 min. During the heat treatment a weight loss of 0.04 wt % was observed in the pure powder with a weight loss of 5.3 wt % in the oxidized powder. This indicates that at least part of the oxide phase evaporated during the heat treatment. Nevertheless, strong differences between the powders were observed. The micrographs of the powder surfaces after heat treatment are shown in Figure 1.

**Figure 2:**
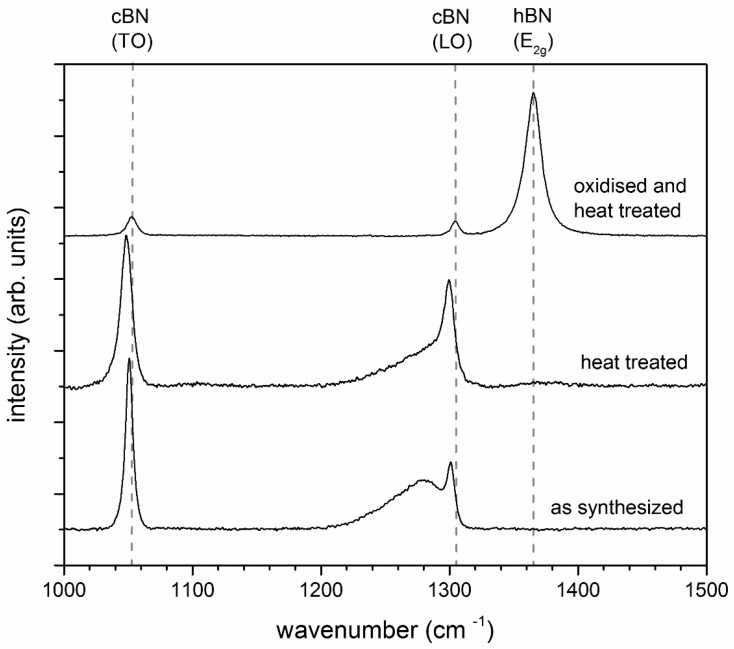
Raman spectra of the two heat treated c-BN powders compared to the c-BN powder as received (synthesized), showing clear differences in the transformation behavior.

The pure powder, without any pre-treatment, did not show evidence of h-BN formation (Figure 1a,b). The powder that was oxidized prior to heat treatment at 1550 °C in Ar exhibited a pronounced transformation to h-BN, which can be identified by spherical outgrows on the surface of the c-BN particles (Figure 1c,d). This was confirmed by Raman investigations of the heat-treated powders and the as-synthesized c-BN powder (Figure 2).

Besides the main band of c-BN at 1306 cm^−1^ [29], a broad shoulder at 1290 cm^−1^ was detected for the heat-treated c-BN powder. The same shoulder was also found in the starting material and was therefore not caused by heat treatment. The shoulder could probably be attributed to the presence of excess boron in the boron nitride grains [29]. The oxidized and heat-treated c-BN powder showed an intense h-BN peak at 1365 cm^−1^ [30] and only small c-BN peaks, indicating extensive transformation of c-BN to h-BN. The results reveal that the liquid phase, especially the amount of boron oxide at the c-BN particle surface, plays a decisive role in the phase transformation. The acceleration of the c-BN → h-BN transformation by B_2_O_3_ could be caused by solution precipitation mechanisms or by gas phase transport.

### 3.2. Heat Treatment of the c-BN–Si_3_N_4_ Composite with and without a Liquid Phase

**Figure 3:**
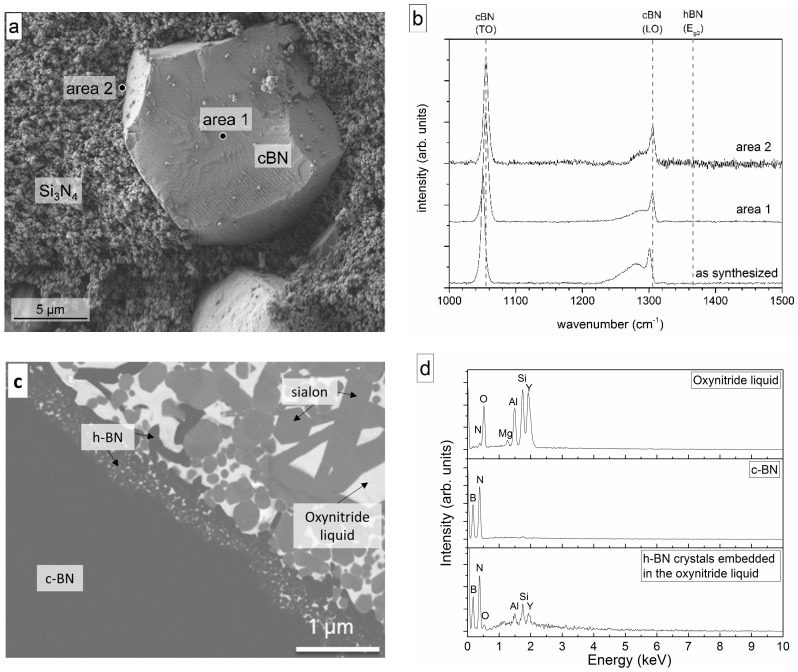
(**a**) SEM micrograph of the fracture surface of the in the spark plasma sintering (SPS) at 1575 °C sintered Si_3_N_4_–c-BN composite without sintering additives and (**b**) Raman spectra of the as-synthesized c-BN powder and one c-BN grain on the fracture surface of the Si_3_N_4_–c-BN composite; (**c**) Interface between c-BN and sialon matrix-densified at 1575 °C. At the interface, the hexagonal boron nitride (h-BN) grains embedded in the amorphous oxynitride grain boundary phase are clearly visible (light gray phase-sialon, bright phase-oxynitride liquid; dark gray-c-BN/h-BN) and (**d**) typical EDX data of these phases.

The c-BN mixed with Si_3_N_4_ without additional additives was sintered at 1575 °C for 5 min in the SPS system. Figure 3 shows the fracture surface of the c-BN–Si_3_N_4_ composite and the Raman spectra of the fracture surface of the composite after sintering in comparison with the as-synthesized c-BN powder. Under these conditions, no h-BN was observed at the interface or on the c-BN surface. This behavior is different from the transformation observed in the SPS densification of the different silicon nitride– and sialon–c-BN composites containing liquid phases at temperatures above 1450 °C [12,13,19].

Besides the main c-BN band (LO-Band of c-BN, see Figure 3b), a broad shoulder, which was also detected in the synthesized c-BN powder, was observed. This shoulder was caused by the presence of excess boron in the boron nitride grains [29], not the heat treatment step. Therefore, it reveals that no h-BN was formed.

The results indicate that Si_3_N_4_ does not accelerate the transformation and thus confirm the data reported by Zhang [2]. However, in composites containing sintering additives used to form the liquid phase required for densification, the stability is much lower.

Figure 3c shows the microstructure of the c-BN–sialon composite densified under the same conditions as the c-BN Si_3_N_4_ powder mixture. The composite containing 30 vol % c-BN and the sintering additives was completely densified due to the liquid phase sintering (Figure 3c). The sialon matrix consisted of crystalline α-sialon, a small amount of ß-sialon phases, and some residual α-Si_3_N_4_. The α-Si_3_N_4_ could be attributed to non-reacted starting powder. Due to the excess Y_2_O_3_, an oxynitride glass also formed at the triple junctions between the sialon and the Si_3_N_4_ grains. Details of the microstructure of sialons are given by Garrett et al. [12,13,14] or more generally by Mandal and Ekström [31,32].

An h-BN layer embedded in the oxynitride amorphous phase, which was a liquid during sintering, was clearly visible at the interface between c-BN and the dense matrix (Figure 3c). Nanocrystalline h-BN could be found at the interface with larger grains farther away from the interface inside the matrix, indicating transport of h-BN during sintering. This was confirmed by Raman spectroscopy (see also [12,13,14]).

### 3.3. Heat Treatment of c-BN–Glass Composites

The c-BN–glass composites were heat-treated at different temperatures between 1100 °C and 1400 °C with a holding time at the peak temperature of 1 h or 10 h respectively. The results of the phase compositions and the determined h-BN content of the glass–c-BN composites for the different heat treatment conditions are given in Table 2 and Table 3.

After heat treatment at 1400 °C all of the samples showed signs of complete melting of the matrix. The samples all had a droplet-like shape and were completely densified. At lower temperatures (<1300 °C), the G2-BN samples, containing G2 and 30 vol % c-BN, did not form droplets. This indicated that fast crystallization took place. It prevented a viscous flow. This was in agreement with the XRD results (Table 2), which showed the existence of a large amount of crystalline phases and a crystallization temperature of 1180–1200 °C, as found by other authors [27,33] for this glass. Similar behavior—i.e., pronounced crystallization (see Table 2)—was observed for G1-BN below 1300 °C.

**Figure 4:**
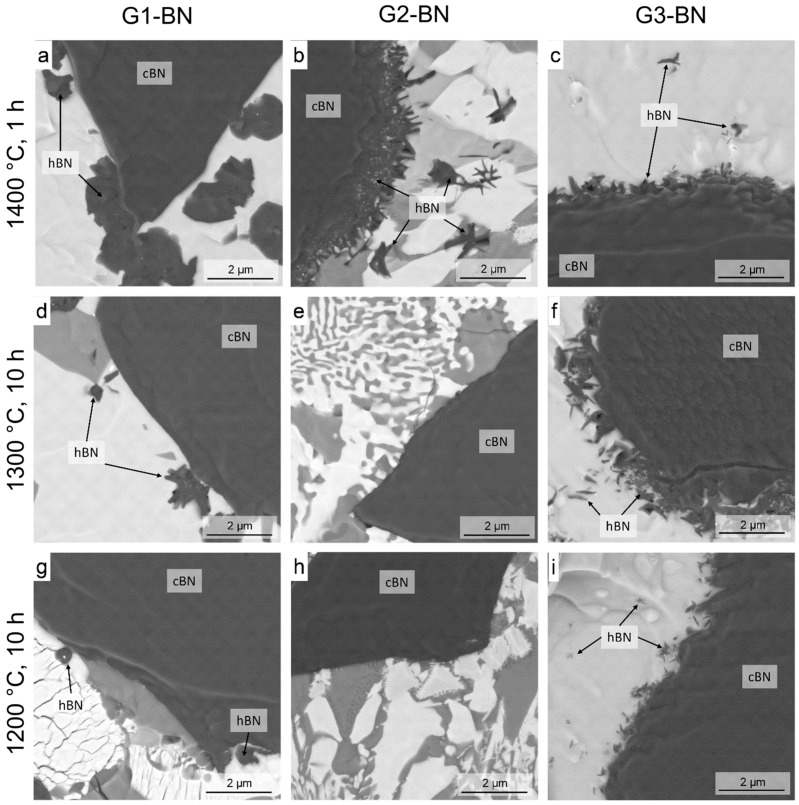
FESEM micrographs of the ion beam polished sections of the glass–c-BN composites heat treated at: (**a**–**c**): 1400 °C, 1 h; (**d**–**f**): 1300 °C; 10 h and (**g**–**i**): 1200 °C, 10 h. From left to right: G1-BN, G2-BN, G3-BN.

The micrographs of the ion beam-polished sections of the composites heat-treated at 1400–1200 °C are given in Figure 4.

In all three compositions, the formation of h-BN was observed and confirmed by EDX measurements and XRD phase analysis after heat treatment at 1400 °C for 1 h (Table 3, Figure 4a–c). The shape and the amount of the h-BN phase formed differed between the compositions. While in G1-BN, the h-BN had a ball-like shape inside the matrix (Figure 4a); in the other two compositions (G2-BN and G3-BN), the h-BN crystals took the form of smaller, more elongated grains at the c-BN–glass interface.

In sample G2-BN, no h-BN was observed at temperatures below 1400 °C. Under these heat treatment conditions, the glass was nearly completely crystallized (Table 2), resulting in minimal SiO_2_-rich glassy phase with a high viscosity. No signs of c-BN transformation were observed in the ion-beam polished sections or by X-ray diffraction also after prolonged heat treatment at lower temperatures in this composition.

The other two composites G1-BN and G3-BN also showed no signs of phase transformation after a 1 h heat treatment at 1300 °C. However, a 10-h heat treatment resulted in phase transformation for these compositions. This transformation was observed by both methods—microstructural analysis in the FESEM (Figure 4d,f) and X-ray diffraction (Table 3).

During heat treatment at 1200 °C for 10 h the phase transformation was also observed only for composites G1-BN and G3-BN, but the transformation was much less pronounced than it was during heat treatment at 1300 °C and 10 h holding time (Figure 4g,i, Table 3). Only 0.6 wt % of h-BN/(h-BN + c-BN) was observed for G1-BN and G3-BN for a heat treatment at 1200 °C and a holding time of 10 h by XRD and FESEM. This was more than ten times less than the amounts of h-BN/(h-BN + c-BN) observed at 1300 °C 10 h (Table 3), suggesting that h-BN was still the stable phase, but the transformation was retarded kinetically. During heat treatment at 1100 °C for 10 h no h-BN formation was observed in any of the c-BN–glass composites.

## 4. Discussion

The results of the investigations reveal that the stability of c-BN is strongly reduced in the presence of a liquid oxide phase:

- Heat treatment of pure c-BN in an argon atmosphere yielded no transformation. In contrast, c-BN showed pronounced transformation to h-BN in the presence of B_2_O_3_ under the same conditions (Section 3.1).
- c-BN showed no conversion in pure Si_3_N_4_ powder at 1575 °C in the absence of a liquid phase, but transformation took place if an oxynitride liquid was formed in the presence of sintering additives (Section 3.2). The latter was also observed in previous experiments performed by the present authors [12,13,14].
- Heat treatment resulted in a much higher c-BN to h-BN transformation rate for mixtures of c-BN and oxide glasses of different compositions than for pure BN, even at 1400 °C and below. This transformation was influenced by the glass composition (Section 3.3).

The experimental results thus clearly show that the transformation is accelerated by the presence of the liquid oxide or oxynitride phases. The mechanisms involved may be different for the different experiments.

### 4.1. Gas Phase Mechanism

In the case of heat treatment of the oxidized and nonoxidized c-BN powders, the effect of the gas phase cannot be completely neglected. Sachdev et al. [22] performed a similar heat treatment experiment, but they did not discuss the influence of B_2_O_3_. They suggested two mechanisms: solid phase transformation and gas phase transport by evaporation of BN. They suggested that a BN pressure of 1 mbar at 1300 °C (data determined in the 1920–1940 years) was enough for transport.

In the present experiments, the observed weight loss was only 0.04% for the pure powder and nearly 5.2%, at least corresponding to the majority of the B_2_O_3_ existing in the samples, for the oxidized powder. Evaporation took place in the temperature range above 1300 °C. This may also be caused by the interaction of the B_2_O_3_ with the carbon crucible used in the experiments. However, no B_4_C was observed in the final powder. The only phases found by XRD after heat treatment were c-BN and h-BN in the oxidized powder and c-BN in the pure starting powder. Despite the evaporation of B_2_O_3_ at high temperatures, the oxide-containing c-BN powder, unlike the material without B_2_O_3_, showed pronounced transformation (Figure 1).

**Figure 5:**
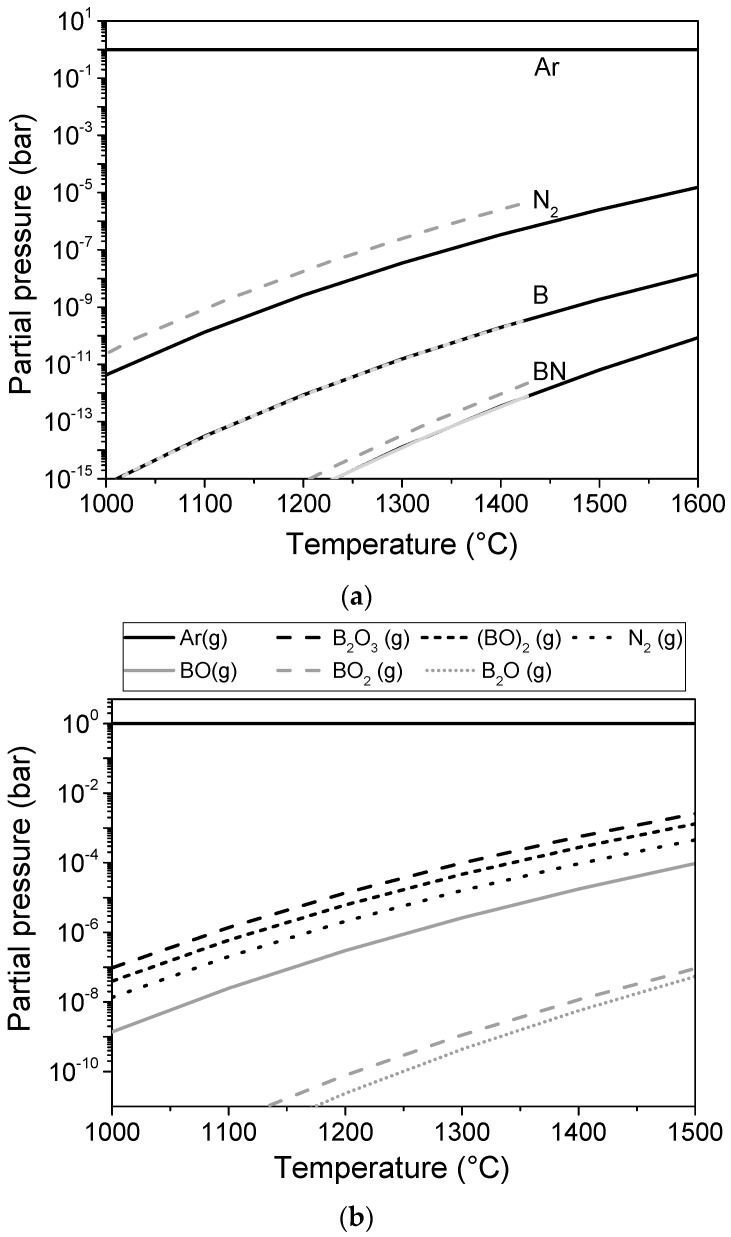
Calculated partial pressures of gas species over h-BN and c-BN (**a**) and h-BN/B_2_O_3_ (**b**); (The dotted lines in Figure 5a correspond to c-BN as a solid phase).

If gas phase transport is involved, transport is proportional to the partial pressure of the species. Figure 5 shows the thermodynamically calculated compositions of the gas phases over h-BN, c-BN, and h-BN/B_2_O_3_. The data were calculated using FACTSAGE 7.1 [34]. The data used for c-BN are given in the [appendix. The partial pressure over pure BN is obviously very low.

BN evaporates incongruently by the following reaction:

(1) $$2\mathrm{BN}\;\to \;2\;B\left(s\right)\;+{\;N}_{2}$$

i.e., it forms gaseous N_2_ as well as solid B at the investigated temperatures. For hexagonal BN, these low evaporation rates were also found experimentally [35]. The data reveal that the actual evaporation rate is nearly 200 times lower than the rate predicted by the thermodynamically calculated partial pressures. Therefore, acceleration of the transformation by the gas phase in the oxygen-free atmosphere at temperatures up to 1600 °C is not likely. This is in agreement with the high stability of the c-BN powder in pure Ar in the absence of an oxide phase observed in the present experiments.

In contrast, for the mixture of BN and B_2_O_3_, the partial pressures of B_2_O_3_, (BO)_2_, and BO are on the same order of magnitude as that of N_2_. Therefore, transport can take place at a similar rate for both boron and nitrogen. Therefore, an accelerated transport can take place. These thermodynamic calculations can explain the observed acceleration of the phase transformation in the presence of an oxide phase. However, as shown by Sachdev, c-BN even reacts with liquid B_2_O_3_ at 850–900 °C [23]. Therefore, an additional mechanism involving liquid B_2_O_3_ cannot be excluded in this case. This will be discussed in more detail in the next section.

The shape of the h-BN formed in the case of the heat-treated c-BN with B_2_O_3_ is different from the shape of the h-BN formed in the glassy phases (Figure 1 and Figure 3). This may be an indication of the involvement of the gas phase transport mechanism in the investigated c-BN–B_2_O_3_ mixture.

### 4.2. Liquid Phase Mechanism

For the mixtures of c-BN and glasses, the effect of the gas phase can be neglected because the glass completely covers the c-BN grains, even at very low temperatures. This was also observed for the composites consisting of c-BN with sialon as the matrix in the presence of a liquid phase. Densification takes place at temperatures at which the c-BN transformation rate is still low [12,13,14]. Therefore, the liquid phase has to take part in the transformation in this case. The main question concerning this mechanism is whether or not BN is soluble in oxide liquids.

As mentioned previously [23], Sachdev and coworkers observed the dissolution of c-BN crystals in the melt at 850–900 °C. After a short time roughening of the surface took place and after 20 h nearly total decomposition of the c-BN took place.

Unfortunately, no phase diagram exists for BN–B_2_O_3_ at ambient pressure, but the diagram has been investigated at 5 GPa [36]. At 1927 °C, a melt with a nitrogen content of between 10 and 17 at % in equilibrium with c-BN has been observed. This corresponds to nearly equimolar amounts of BN, B_2_O_3_, and B in the melt, indicating a fairly high solubility of c-BN. This solubility decreases with reduction of temperature.

The effect of oxygen/B_2_O_3_ on the phase transformation of h-BN to c-BN at high pressures has been studied extensively [37,38,39,40]. Choi et al. [37] showed that amorphous or crystalline h-BN transforms to c-BN at a much lower rate in the absence of B_2_O_3_ at 4.6 GPa and 1350 °C (AlN-catalyzed). Similar results with a Mg–Al alloy catalyst were obtained by Singal et al. [39]. In a study performed by Gladkaya et al. [40], activation of the transformation by an ammonium borate melt was observed. However, crystallization of a solid oxide phase such as MgO may hinder crystallization of c-BN due to oxide impurities [38]. These data indicate that the oxide melt can have a strong influence on the h-BN → c-BN transformation and suggest that the reverse transformation can also be influenced.

A few studies have been published on the equilibrium solubility of h-BN in B_2_O_3_ [41,42], B_2_O_3_-containing melts (B_2_O_3_–SiO_2_ and B_2_O_3_–CaO) [41], and borate melts containing alkalis, alkaline earths, and Y_2_O_3_ [42]. In all of them, a solubility of h-BN was reported. The solubility of h-BN in pure B_2_O_3_ was found to be 1.1 wt % at 1550 °C, 0.28 wt % at 1300 °C, and 0.05 wt % at 1100 °C. In a melt containing 50 mol % B_2_O_3_ and 50 mol % SiO_2_, a solubility of 0.15 wt % was observed.

The solubility of BN increases with increasing amount of alkaline and earth alkaline oxides two to three times in comparison to a pure B_2_O_3_ melt at 1200 °C [42]. The solubility is nearly constant in B_2_O_3_ melts containing 0–20 mol % Al_2_O_3_ or Y_2_O_3_ at 1550 °C [42]. For B_2_O_3_–Na_2_O glasses with 15 mol % Na_2_O, Frischat obtained a solubility of nitrogen of 2.2 wt %, corresponding to 3.8 wt % BN, at 1150 °C [43].

Based on these data, a solubility of at least 0.1–0.01 wt % h-BN has to be considered in the different oxide melts under the conditions used in the present experiments. This seems sufficient to enable the slow dissolution of c-BN and precipitation of h-BN in the liquid.

The morphology of the h-BN formed in the glasses (Figure 4a–c) shows that the grains are formed outside of the original BN grains and have a shape that depends on the composition of the glass. In glass G1, relatively large rounded grains are formed; whereas in the other two glasses, elongated grains with the main axes perpendicular to the surface are formed. This indicates the influence of the nature of the melt (solubility, interfacial energy, diffusion rate) on the grain shape. Such effects were explained for other materials in [44,45].

In principle, formation of the nucleus by direct solid-state c-BN–h-BN transformation can take place. However, it seems to be very unlikely in the investigated systems. In earlier TEM investigations conducted by the authors [13], no direct c-BN–h-BN conversion on the surface was observed in c-BN–sialon composites. In all cases, a thin Y-rich layer was found between the c-BN grain and the formed h-BN nucleus. Additionally, in the system Si_3_N_4_–c-BN and the pure c-BN, h-BN was not observed on the surface of c-BN if no liquid was formed. This is additional evidence of the very low direct transformation rate under the present experimental conditions.

These findings are in agreement with the data published by Zhang [2,11]. c-BN shows a high stability in mixtures of c-BN and nonreacting components such as TiN and Si_3_N_4_.

Very recent results of sintering of Ca-sialon at 1500 °C only showed formation of h-BN for 20 µm c-BN in the presence of a suitable amount of oxynitride liquid [21].

The stability of c-BN in contact with an oxide or oxynitride liquid is much lower than the literature data suggest [2,11]. The results of the heat treatment of G1-BN and G3-BN showed that at temperatures as low as 1200 °C, the conversion of c-BN was still observed, indicating that at least at these temperatures > 1200 °C, the h-BN is the thermodynamically stable phase.

**Figure A2:**
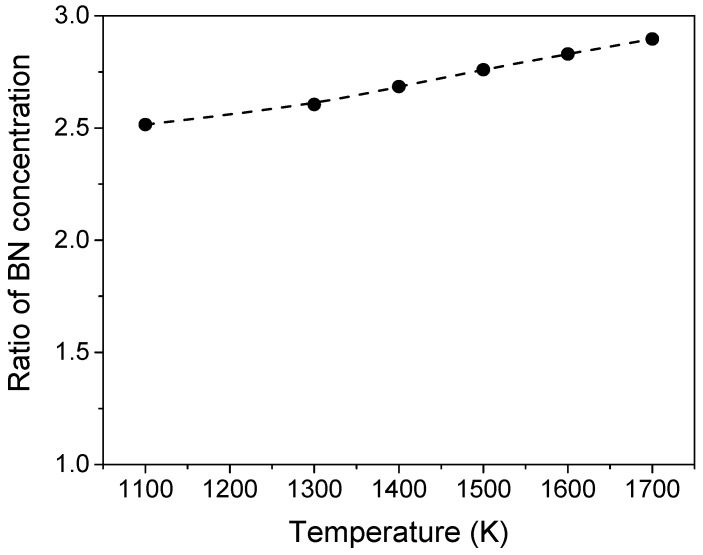
Temperature dependence of the ratio of the equilibrium concentrations of BN in the melt over c-BN and h-BN calculated based on Equation (A7) and ΔG° (cBN/hBN) (see Figure A1).

Based on these experimental observations, the following mechanism for the c-BN–h-BN conversion can be proposed. The conversion is most probably controlled by a solution–diffusion–precipitation mechanism. The c-BN slowly dissolves in the oxide or oxynitride liquid. A certain solubility of boron nitride in oxide and oxynitride liquids has been reported in the literature, as mentioned previously. The solubility is the highest in pure B_2_O_3_ and increases with temperature. As can be shown by thermodynamic calculations, the equilibrium solubility of c-BN is even higher than that of h-BN (Appendix A). In the temperature region of interest, the ratio of the equilibrium solubilities of c-BN and h-BN in the melt can be estimated to be 2.5–3; i.e., the equilibrium solubility of c-BN is nearly three times that of h-BN (see Appendix A, Figure A2). After the solubility limit of boron and nitrogen in the liquid is reached at least near the dissolving c-BN grains, h-BN nucleation begins and h-BN grains crystallize. The h-BN formation will take place near the dissolving c-BN grains because of the low diffusion coefficients in the silicate melts and the absence of convection.

Due to the different equilibrium solubilities of c-BN and h-BN, a concentration gradient of boron and nitrogen ions exists between the surfaces of the c-BN and h-BN particles in the melt. The conversion rate therefore depends either on the dissolution rate—which is most likely lower for well-grown c-BN particles with larger grain sizes—or on the diffusion in the glass for fine-grained materials at low temperatures or in viscous melts.

In the temperature range of interest, the differences between the saturation concentrations of c-BN and h-BN only change slightly, although the total solubility can change quite significantly, as the experimental data for solubility of h-BN in B_2_O_3_ have shown [41,42]. Additionally, there is a strong correlation between the diffusion coefficient and the viscosity. Hence, different h-BN conversion rates are found for the different composites at different temperatures (Table 3). The interpretation of the different conversion rates in G1-BN and G3-BN composites is difficult because the conversion rate depends on the viscosity, the BN solubility, and the crystallization behavior of the glasses. These parameters are difficult to quantify and no reliable data for them exist in the literature. Hence, the discussion below will focus on the behavior of the G2-BN composite.

It has shown no transformation <1300 °C and a similar rate at 1400 °C as the other two composites. The reason for this seems to be the low stability of this glass in comparison with the other compositions. The glass in G2-BN crystallizes quite rapidly, even during heating, as was shown by other authors [27,33]. Therefore, below the eutectic temperature of 1371 °C, only a very limited amount of residual glass exists due to the fact that the crystallization in these systems is not complete. This residual glass is very rich in SiO_2_ [27,33] and has a high viscosity and a reduced solubility of h-BN. Hence, the conversion is strongly retarded in comparison with the heat treatment at 1400 °C and the conversion rates of c-BN embedded in the other two composites G1-BN and G3-BN with the more stable glasses.

The conversion is directly connected with the T-p phase diagram of boron nitride, which is still under discussion [46,47,48]. Data of Solozhenko [10,47] reveal that c-BN is the stable phase for T ≤ 1300 °C at ambient pressure. A conversion temperature at ambient pressure below 300 °C [48] and even 0 K [9] can be extrapolated based on high-pressure experiments. The first time that the conversion was observed at ambient pressure at a temperature of 1200 °C in oxide liquids was in the present experiments. These experiments reveal that at ambient pressure and at temperatures of at least 1200 °C, h-BN is the stable modification. These data agree with the findings of Fukunaga et al. [9] and Will et al. [48], but not with the data published by Solozhenko [47].

The observed lowest conversion temperature of 1200 °C seems to be controlled by kinetics and is not the thermodynamic stability limit. The results reveal that the transformation of c-BN can only be suppressed kinetically.

To suppress the c-BN–h-BN transformation in c-BN ceramic matrix composites during sintering above 1200 °C, it is necessary to ensure that no liquid phase that can dissolve BN is in direct contact with the surface of c-BN or, if it is, to ensure that the viscosity of the liquid phase is high enough to prevent the phase transformation. The transformation can even be triggered by the formation or existence of B_2_O_3_ on the c-BN surface. B_2_O_3_ forms low-viscosity liquids with many oxides. Hence, B_2_O_3_ surface layers on the starting c-BN particles accelerate the transformation, for example, in an Al_2_O_3_ or TiN matrix.

The observed decrease in the extent of transformation due to SiO_2_ coatings on c-BN [20] can be explained by this mechanism. The viscosity of the amorphous SiO_2_ phase is quite high and the solubility of BN in pure SiO_2_ is very low [41]. In contrast, for c-BN powders with B_2_O_3_ surface layers or alkali or alkaline earth impurities on the surface, the transformation is strongly accelerated. Besides the solution–precipitation mechanism, gas phase reactions occurring due to the high B_2_O_3_ partial pressure (especially in the presence of moisture) may cause the acceleration of the transformation by B_2_O_3_.

A B_2_O_3_ surface layer on c-BN can also explain h-BN formation in non-oxide materials such as TiN, AlN, and Si_3_N_4_ because the B_2_O_3_ reacts with the nitrides and forms h-BN and the oxide of the matrix element, resulting in formation of an at least transient oxynitride liquid, which accelerates the transformation.

## 5. Conclusions

The experiments involving heat treatment of c-BN particles in oxide glasses with different compositions in the range of 1100–1400 °C demonstrated that the c-BN–h-BN conversion could take place at much lower temperatures than those proposed in the literature [10,46,47]. Even at 1200 °C, the transformation of c-BN to h-BN was observed in the presence of a B_2_O_3_-containing glassy phase with a sufficiently low viscosity. The most probable transformation mechanism that could explain all of the experimental data was found to be dissolution of the metastable c-BN in the glass or melt, and diffusion and precipitation of the stable h-BN. Therefore, this process should take place in ceramic-matrix composites in which a liquid phase dissolving h-BN exists during densification. Even the existence of small amounts of B_2_O_3_ on the surface of the c-BN resulted in the formation of (at least) a transient liquid (due to the low-melting eutectic with a wide range of oxides), in turn resulting in the formation of thin h-BN layers. This causes the weak bonding of c-BN in the matrix often observed. As a consequence, these materials exhibited lower hardness and wear resistance than expected.

The influence of metal melts on the transformation has not yet been investigated. If the melt can dissolve BN, then a similar mechanism can be possible. However, there is a lack of data concerning the solubility of c-BN in liquid metals. The use of h-BN as a refractory material for different metal melts indicates that the solubility might be much lower than in oxide or oxynitride glasses and therefore a higher stability could be expected.

The existence of B_2_O_3_ on the surface of BN could be a reason for the reduced stability of fine-grained c-BN grains even under inert conditions. The finer grain size and the higher surface area result in a higher B_2_O_3_ content than that of coarse-grained c-BN particles, and the pore channels of the powder pile or pressed body are finer. Therefore, the evaporation of B_2_O_3_ is not fast enough to prevent the transformation. Additionally, the evaporation of B_2_O_3_ strongly depends on the moisture content [49,50]. 

In samples containing c-BN and low amounts of B_2_O_3_ and with high porosities at high temperatures, the gas phase can also accelerate the cubic-to-hexagonal phase transformation. This transformation must be sensitive to the B_2_O_3_ content because the gas pressure of the boron-containing species is several orders of magnitude higher in a B_2_O_3_/BN mixture than in oxide-free c-BN.

## Figures and Tables

**Table 1:** Compositions and glass transition temperatures of the glasses used.

Sample	Glass Composition (wt %)							T_g_ (°C)
	SiO_2_	B_2_O_3_	Al_2_O_3_	MgO	CaO	BaO	Y_2_O_3_	
G1 ^1^	19.9	13.2	24.1	-	-	-	42.8	740
G2 ^2^	15.6	-	20.5	-	-	-	63.9	890
G3 ^3^	59.6	5.3	15.6	2.5	7.7	9.4	-	725

^1^ G1 was produced by melting at 1650 °C at IKTS Dresden; ^2^ G2 was provided by TU Dresden; ^3^ Data for G3 from [25].

**Table 2:** Phase composition of the glass matrix of the glass–c-BN composites as a function of heat treatment conditions determined by XRD measurement.

Temperature (°C)	Holding Time (h)	Phase Composition		
		G1-BN	G2-BN	G3-BN
1400	1	Amorphous	Mullite α-Y_2_Si_2_O_7_ γ-Y_2_Si_2_O_7_	Amorphous
1300	1	Y(Al)BO_3_ (α-Al_2_O_3_) Al_9_BSi_2_O_19_	Mullite Y_2_Si_2_O_7_ YAG SiO_2_	Amorphous
1300	10	YBO_3_ (α-Al_2_O_3_) Al_9_BSi_2_O_19_	Mullite Y_2_Si_2_O_7_ YAG SiO_2_	Amorphous
1200	10	YBO_3_ Al_9_BSi_2_O_19_ α-Y_2_Si_2_O_7_ γ-Y_2_Si_2_O_7_	Mullite, YAG Y_2_Si_2_O_7_, SiO_2_ α-Y_2_Si_2_O_7_ γ-Y_2_Si_2_O_7_	Amorphous
1100	10	YBO_3_ α-Y_2_Si_2_O_7_ γ-Y_2_Si_2_O_7_	Not investigated	SiO_2_

**Table 3:** Heat treatment conditions and results of analysis of h-BN formation based on microstructural analysis and XRD investigations.

Temperature (°C)	Holding Time (h)	Content of h-BN/(h-BN+c-BN) (wt %)		
		G1-BN	G2-BN	G3-BN
1400	1	h-BN (24.6%)	h-BN (13.7%)	h-BN (12.2%)
1300	1	No h-BN	No h-BN	No h-BN
1300	10	h-BN (10.2%)	No h-BN	h-BN (6.7%)
1200	10	h-BN (0.6%)	No h-BN	h-BN (0.6%)
1100	10	No h-BN	Not investigated	No h-BN

**Figure A1:**
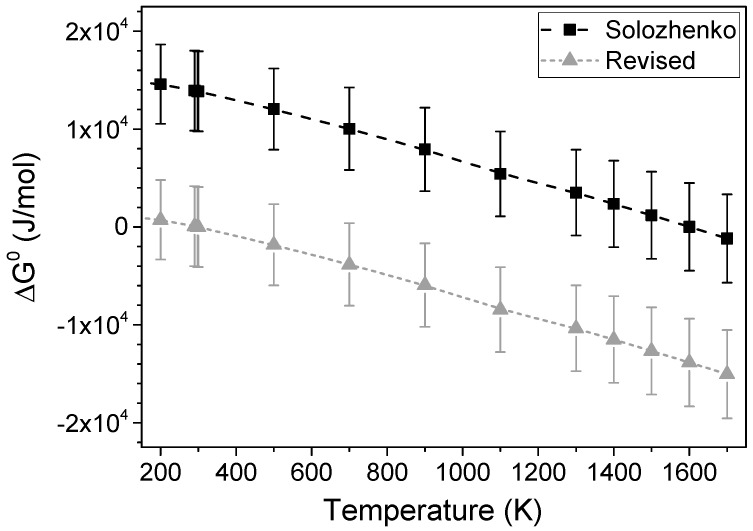
Gibbs free energy ΔG° of the c-BN to h-BN transformation.

## Appendix A

The thermodynamic data of Solozhenko and the derived phase diagram reveal that c-BN is the stable phase up to 1300 °C at ambient pressure [22,46,47]. However, the extrapolation of more recent c-BN–h-BN phase transformation data to ambient pressure by Will [48] and Fukunaga [9] as well as the experiments in the present work show that h-BN must be the stable phase even below 1300 °C. The data of Will predict the transformation at ambient pressure at approximately 200–300 °C [48] and the data of Fukunaga even at 0 K [9]. At these temperatures, ΔG° (hBN/cBN) of the reaction would be 0.

(A1) cBN → hBN

The approximation that ΔG° of the transformation reaction would be 0 at 298 K was used for the present calculations. The values of S and c_p_ for h-BN and ΔH°(h-BN) = 250.5 ± 2 kJ/mol from Solozenko [46] are in good agreement with the tabulated data for h-BN [34] (less than 0.2% relative error). Therefore, these data and the S and H°(t)–H°(0 K) data for c-BN given in the literature [46] were also used and only the ΔH°(cBN, 298) was adapted to fulfill the condition ΔG°(hBN-cBN, 298) = 0:

(A2) $$\Delta G^{0}\left(298\right)=0=\Delta H^{0}\left(\mathrm{hBN},298\right)\;‒\;\Delta H^{0}\left(\mathrm{cBN},298\right)-298K\cdot \left(S\left(\mathrm{hBN},298\right)\;‒S\left(\mathrm{cBN},298\right)\right)$$

This results in the following equation:

(A3) $$\Delta H^{0}\left(\mathrm{cBN},\;298\right)\;=\;\Delta H^{0}\left(\mathrm{hBN},\;298\right)\;‒\;298K\cdot \left(S\left(\mathrm{hBN},298\right)\;‒\;S\left(\mathrm{cBN},298\right)\right)$$

This result in the shift of ΔH°(c-BN) from 266.8 kJ ± 2.2 kJ/mol (given in the literature [46]) to 252.9 kJ/mol.

Based on these data, the ΔG° of reaction (A1) as a function of temperature is expressed in Figure A1. Given the same error as observed for the original data, the data would agree with the observed phase transformation of Will and Fukunaga.

Therefore, these data were used for estimating the partial pressures of the different gases and the solubility of c-BN in the melt.

### Solubility of c-BN/h-BN

If it is assumed that the melt is at least locally in equilibrium at the c-BN–oxynitride liquid interface, the chemical potential of BN in both phases must be equal:

(A4) $$\mu \left(\mathrm{cBN},\;\mathrm{solid}\right)\;=\;\mu (\mathrm{BN},\;\mathrm{liquid})\;=\;\mu_{0}\left(\mathrm{BN},\;\mathrm{liquid}\right)\;+\mathrm{RT}\;\ln\left(a\left(\mathrm{cBN}\right)\right)$$

The chemical potential of c-BN is given by μ(cBN, solid) and the chemical potential in the liquid is given by μ(cBN, liquid). μ_0_(BN, liquid) is the standard potential of BN in the liquid; a(cBN) and a(hBN) is the activity of BN in the liquid in equilibrium with c-BN and h-BN correspondingly. The activity is proportional to the product of concentration of BN in the melt (c(BN)) and the coefficient of activity (f).

For the surface of a h-BN particle, the local equilibrium can be described in the same way:

(A5) $$\mu (\mathrm{hBN},\;\mathrm{solid})=\;\mu_{0}\left(\mathrm{BN},\;\mathrm{liquide}\right)\;+\mathrm{RT}\;\ln\left(a\left(\mathrm{hBN}\right)\right)$$

Combining Equations (A4) and (A5) yields the following equation:

(A6) $$\mu (\mathrm{cBN},\;\mathrm{solid})-\;\mu (\mathrm{hBN},\;\mathrm{solid})=\;+\mathrm{RT}\;\ln\left(\frac{a\left(\mathrm{cBN}\right)}{a\left(\mathrm{hBN}\right)}\right)$$

This can also be written as follows:

(A7) $$e^{\frac{\Delta G\left(\mathrm{cBN}/\mathrm{hBN}\right)}{\mathrm{RT}}}=\frac{f\cdot c\left(\mathrm{cBN}\right)}{f\cdot c\left(\mathrm{hBN}\right)}$$

Using the data from the transformation c-BN to h-BN and assuming the coefficient of activity f = const. at the given temperature, it is possible to obtain the temperature dependence of the ratio of the equilibrium solubilities using Equation (A7) (Figure A2). The data reveal that the c-BN solubility in equilibrium is 2.5 to 3 times as high as the solubility of h-BN in the temperature region of 1100 to 1700 K. Using the determined solubility of h-BN in B_2_O_3_ [41] at 1300 °C of 0.28 wt %, it is possible to calculate the solubility of c-BN at 1300 °C (1573K) as 2.8×0.28 wt % = 0.78 wt %. This value is sufficient for a sufficiently fast solution–precipitation mechanism.

The ratio of equilibrium saturation concentrations of c-BN and h-BN in the liquid is directly connected with the change in the Gibbs free energy (ΔG°) of the reaction for conversion of c-BN to h-BN at the given temperature. The ratio decreases with decreasing difference in the Gibbs free energy and becomes 1 if c-BN stops being the metastable phase (at 298 K in the present calculations).

The chemical potential also depends on the grain size. Very fine-grained h-BN particles compensate at least partially for the differences in the chemical potential. Therefore, a critical h-BN size of nucleus is necessary for the transformation.
